# A Nomogram for Predicting Intraoperative Hemodynamic Instability in Patients With Pheochromocytoma

**DOI:** 10.3389/fendo.2021.787786

**Published:** 2022-01-06

**Authors:** Zhiqiang Zhang, Yunlin Ye, Jiajie Yu, Shufen Liao, Weibin Pan, Yan Guo, Shuangjian Jiang, Cheng Luo, Fufu Zheng

**Affiliations:** ^1^ Department of Urology, First Affiliated Hospital, Sun Yat-Sen University, Guangzhou, China; ^2^ Department of Urology, Cancer Center, Sun Yat-Sen University, Guangzhou, China; ^3^ Department of Anesthesia Surgery Center, First Affiliated Hospital, Sun Yat-Sen University, Guangzhou, China; ^4^ Department of Radiology, First Affiliated Hospital, Sun Yat-Sen University, Guangzhou, China

**Keywords:** pheochromocytoma, hemodynamics, nomograms, tomography, X-ray computed, surgery

## Abstract

**Purpose:**

Surgical removal of pheochromocytoma (PCC), including open, laparoscopic, and robot-assisted adrenalectomy, is the cornerstone of therapy, which is associated with high risk of intraoperative and postoperative life-threatening complications due to intraoperative hemodynamic instability (IHD). This study aims to develop and validate a nomogram based on clinical characteristics as well as computed tomography (CT) features for the prediction of IHD in pheochromocytoma surgery.

**Methods:**

The data from 112 patients with pheochromocytoma were collected at a single center between January 1, 2010, and December 31, 2019. Clinical and radiological features were selected with the least absolute shrinkage and selection operator regression analysis to predict IHD then constitute a nomogram. The performance of the nomogram was assessed in terms of discrimination, calibration, and clinical utility.

**Results:**

Age, tumor shape, Mayo Adhesive Probability score, laterality, necrosis, body mass index, and surgical technique were identified as risk predictors of the presence of IHD. The nomogram was then developed using these seven variables. The model showed good discrimination with a C-index of 0.773 (95% CI, 0.683–0.862) and an area under the receiver operating characteristic curve (AUC) of 0.739 (95% CI, 0.642–0.837). The calibration plot suggested good agreement between predicted and actual probabilities. Besides, calibration was tested with the Hosmer–Lemeshow test (P = 0.961). The decision curve showed the clinical effectiveness of the nomogram.

**Conclusions:**

Our nomogram based on clinical and CT parameters could facilitate the treatment strategy according to assessment of the risk of IHD in patients with pheochromocytoma.

## Introduction

Pheochromocytoma (PCC) is a rare catecholamine-producing tumor arising from the chromaffin cells in the adrenal medulla. The incidence is about 0.3–0.5 cases per 100,000 person-years (1, 2). Excessive secretion of catecholamines (CAs) (epinephrine, norepinephrine, and dopamine) may evoke typical manifestations such as sustained or episodic hypertension, headaches, palpitations, and perspiration. However, it is not an unusual phenomenon that pheochromocytomas were discovered as adrenal incidentalomas with the widespread use of cross-sectional imaging for unrelated disorders.

Since surgical removal of pheochromocytoma is the cornerstone of therapy, intraoperative hemodynamic instability (IHD), which could cause intraoperative and postoperative catecholamine-induced life-threatening complications, is the major concern of a multidisciplinary team. Despite a number of previous studies, the predictive risk factors associated with IHD during pheochromocytoma resection remain inconclusive (3–7). In the present study, the aim was to develop and validate a nomogram that incorporated multiple preoperative radiological and clinical parameters for individual preoperative prediction of hemodynamic instability during the surgery of pheochromocytoma.

## Materials and Methods

### Ethics Statement

This retrospective study was approved by the Institutional Review Board at the First Affiliated Hospital, Sun Yat-sen University (Guangdong, China). Written informed consent was waived.

### Subjects

The entire cohort of this study consisted of patients with PCC who underwent adrenalectomy with curative intent between January 1, 2010, and December 31, 2019. Patients who underwent unilateral adrenalectomy and were diagnosed with PCC by postoperative histological examination were included. Patients meeting any of the following criteria were excluded from the follow-up analysis: (1) bilateral adrenalectomy or surgery for recurrent PCCs; (2) without preoperative unenhanced and contrast-enhanced computed tomography (CT) records; and (3) incomplete medical data.

### Data Collection

After the screening, two authors independently retrieved the information of enrolled patients from medical records using a uniform schedule. Demographics, clinical manifestations, medical and surgery history, comorbidities, hemodynamics, laboratory results, CT data, and surgical records were derived. Preoperative baseline systolic blood pressure (SBP) and heart rate (HR) were defined as the values recorded in the morning of the surgical intervention.

Additionally, preoperative CT scans were retrospectively retrieved and evaluated by two senior radiologists. From the images, location of lesions, tumor size, mean Hounsfield units (HU), on unenhanced CT and contrast-enhanced CT, necrosis and Mayo Adhesive Probability score (MAP) were considered. Drawing the region of interest (ROI) was performed at the level of maximum diameter of tumor in unenhanced and contrast-enhanced CT and avoided the area of necrosis, cystic degeneration, bleeding, and calcification. Then mean unenhanced and enhanced HU values were calculated for subsequent analysis. The MAP, a simple image-based scoring system ranging from 0 to 5, termed the summed scores of posterior perinephric fat thickness and stranding (8). Necrosis changes were identified as hypodense regions with 0–20 HU on unenhanced studies in the lesions and less than 10 HU increase on enhanced studies (9).

A non-selective alpha-blocker phenoxybenzamine (PXB) was initiated preoperatively in patients with biochemical proof and typical imaging findings of PCC and was titrated according to blood pressure and tolerability. After initiation of PXB, beta-blockade was used in patients with tachycardia. In order to achieve target blood pressure levels, a calcium channel blocker was added in refractory patients. Adequate blockade was defined by a target blood pressure of 130/80 mmHg, a target heart rate of 90 beats/min, and a target hematocrit of 0.45. Robot-assisted, laparoscopic, or open surgeries were performed according to preoperative evaluation of tumor location and size. All surgeries were performed by experienced surgeons. For a surgery of PCC, standard general anesthesia was conducted and an arterial line for monitoring intraoperative hemodynamics was routinely inserted before induction. Intraoperative hemodynamic data including blood pressure, heart rate, and the use of vasoactive dugs were recorded. IHD was defined as at least one intraoperative episode of SBP >200 mmHg, SBP greater than or less than 30% of baseline, or HR > 110 beats per minute (bpm) (10).

### Statistical Analysis

To determine preoperative parameters predicting IHD, we compared the group of patients with IHD to the group without IHD using the chi-squared test for categorical variables. Continuous variables were categorized based on predefined cutoffs of interest. The least absolute shrinkage and selection operator (LASSO) regression analysis was used to identify the significant variables associated with IHD. The predictive values of screened significant variables were measured by the index of the probability of concordance (C-index). A nomogram was constructed on the basis of these selected variables.

The nomogram performance was evaluated with respect to discrimination and calibration. Discrimination of the nomogram was presented as the C-index and the area under the receiver operating characteristic curve (AUC). Moreover, calibration of the nomogram was tested by plotting the observed outcome probabilities and the Hosmer–Lemeshow (H–L) test. Moreover, the bootstrap resampling method was used to perform internal validation. To determine the clinical effectiveness, the nomogram was subjected to decision curve analysis.

Statistical significance was set as a two-sided value of P < 0.05. Statistical analyses were performed using SPSS version 25.0 (IBM SPSS Inc., Chicago, IL, USA), and R software version 3.6.0 (http://www.r-project.org) with the “rms” package.

## Results

### Characteristics of the Subjects

After screening based on the same inclusion and exclusion criteria, 112 patients were enrolled in the development cohort. The demographic, clinical, and CT characteristics of the patients are presented in **Table 1**. Due to discrepancy of biochemical tests, levels of CAs and their metabolites were not included for a further analysis (**Supplementary Table 1**). All patients underwent preoperative alpha-blockade. Overall, 75 (77.0%) patients had IHD. Age, gender, the presence of the classic triad, tumor diameter, mean CT value difference of unenhanced and contrast-enhanced CT, tumor shape, MAP, hematocrit (HCT), previous surgical history, American Society of Anesthesiologists physical status (ASA) score, surgical technique, and preoperative blood pressure did not differ significantly between patients with and without IHD (**Table 1**). Compared with the patients without IHD, the patients with IHD were more likely to harbor low body mass index (BMI) (P <0.05), left-sided tumors (P < 0.05), and presence of necrosis (P < 0.05).

**Table 1:** Clinical characteristics of enrolled patients.

	All patients (n = 112)No. of patients (%)	Without IHD (n = 37)No. of patients (%)	With IHD (n = 75)No. of patients (%)	P value
Age (years)				0.430
≥60	21 (18.8)	3 (14.3)	18 (85.7)	
<60	91 (81.3)	34 (37.4)	57 (62.6)	
Gender				0.956
Female	67 (59.8)	22 (32.8)	45 (67.2)	
Male	45 (40.2)	15 (33.3)	30 (66.7)	
BMI (kg/m^2^)				0.021
>25	20 (17.9)	11 (55.0)	9 (45.0)	
≤25	92 (82.1)	26 (28.3)	66 (71.7)	
Classic symptoms				0.252
Yes	66 (58.9)	19 (28.8)	47 (71.2)	
No	46 (41.1)	18 (39.1)	28 (60.9)	
CT-based features				
Laterality				0.012
Right	63 (56.3)	27 (42.9)	36 (57.1)	
Left	49 (43.7)	10 (20.4)	39 (79.6)	
Diameter (cm)				0.342
>6	37 (33.0)	27 (73.0)	10 (27.0)	
≤6	75 (67.0)	48 (64.0)	27 (36.0)	
Mean CT value difference				0.315
>53	56 (50.0)	21 (37.5)	35 (62.5)	
≤53	56 (50.0)	16 (28.6)	40 (71.4)	
Shape				0.214
Round type	13 (11.6)	7 (53.8)	6 (46.2)	
Lobular type	91 (81.3)	27 (29.7)	64 (70.3)	
Irregular type	8 (7.1)	3 (37.5)	5 (62.5)	
MAP				0.092
0–1	83 (74.1)	32 (38.6)	51 (68.0)	
2	16 (14.3)	2 (12.5)	14 (87.5)	
3–4	11 (11.6)	3 (23.1)	10 (76.9)	
Necrosis				0.049
Yes	48 (42.9)	11 (22.9)	37 (77.1)	
No	64 (57.1)	26 (40.6)	38 (59.4)	
HCT				0.228
<0.45	97 (86.6)	30 (30.9)	67 (69.1)	
≥0.45	15 (13.4)	7 (46.7)	8 (53.3)	
Surgical history				0.750
Yes	20 (17.9)	6 (30.0)	14 (70.0)	
No	92 (82.1)	31 (33.7)	61 (66.3)	
ASA				0.525
I	9 (8.0)	4 (44.4)	5 (55.6)	
II	69 (61.6)	24 (34.8)	45 (65.2)	
III	34 (30.4)	9 (26.5)	25 (73.5)	
Surgical technique				0.201
Open surgery	33 (29.5)	8 (24.2)	25 (75.8)	
Laparoscopic/Robotic-assisted	79 (70.5)	29 (36.7)	50 (63.3)	
Preoperative blood pressure				0.454
Normal	40 (35.7)	15 (37.5)	25 (62.5)	
Hypertensive	72 (64.3)	22 (30.6)	50 (69.4)	

IHD, intraoperative hemodynamic instability; CT, computed tomography; MAP, Mayo Adhesive Probability score; HCT, hematocrit; ASA, American Society of Anesthesiologists physical status.

### Feature Selection and Risk Factor Analysis of IHD

The optimal λ value was chosen in the LASSO model according to 10-fold cross-validation based on the minimum criteria and the one standard error of the minimum criteria (the 1-SE criterion). Features with non-zero coefficients were selected by LASSO analysis (**Supplementary Figures 1A, B**). The predictive values of significant variables are presented in **Supplementary Table S2**.

### Development and Validation of an Individualized Prediction Model for IHD

Model 1 included seven clinical as well as CT variables (i.e., age, tumor shape, BMI, laterality, MAP, necrosis). However, model 2 only included clinical factors age, BMI, laterality, and surgical technique. Compared with model 2 (AUC: 0.694; 95% CI 0.590–0.797), model 1 with the highest AUC (AUC: 0.739; 95% CI 0.642–0.837) was chosen as the final model (**Supplementary Figure 2A**), indicating the importance of incorporating CT features. Besides, the final model showed good discrimination with a C-index of 0.773 (95% CI, 0.683–0.862) and was presented as a nomogram (**Supplementary Figure 2B**). The calibration plot suggested good agreement between predicted and actual probabilities (**Supplementary Figure 3A**). Moreover, calibration was tested with the Hosmer–Lemeshow test (P = 0.961).

### Clinical Use

The decision curve analyzed was plotted to evaluate the clinical effectiveness of the nomogram (**Supplementary Figure 3B**). The plots showed that the use of this nomogram to predict IHD was associated with net benefit gains compared to the treat-all strategy or treat-none strategy.

## Discussion

Special considerations are required for IHD during resection of pheochromocytoma, a functional tumor, as blood-pressure spike and drop are associated with an increased risk of complications. Current evidence shows that age, preoperative blood pressure, orthostatic hypotension, BMI, body weight change, HCT, heart rate, higher catecholamine levels, tumor size, alpha-blockade type, hydration status, and procedure type may be associated with IHD during pheochromocytoma resection (3–7). However, CT, the most widely used and effective imaging modality for the diagnosis of PCC, provides excellent morphologic data but was seldomly used to predict IHD (11). Therefore, we aimed to develop a user-friendly tool that specially incorporates CT characteristics to predict the risk of encountering IHD.

This present study identified seven preoperative predictive factors for intraoperative hemodynamic instability during unilateral adrenalectomy, including age, tumor shape, Mayo Adhesive Probability score, laterality, necrosis, body mass index, and surgical technique. We utilized the seven predictive factors to create a nomogram that predicts the likelihood of the occurrence of IHD.

In our results, age >60 years was an effective predictor for IHD. We speculated that the observed characteristics might be the combined effect of diminished cardiac reserve and increased vascular resistances responding to intraoperative stimulations.

In our study, lobular type and irregular type were associated with IHD with regard to tumor shape. The present analyses showed that round type was frequently found in pheochromocytomas (9, 12). To the best of our knowledge, the shape of lesions was not adopted into reports on risk factors of IHD in patients with pheochromocytoma. Moreover, we speculated that lobular type and irregular type might be the impact of activated angiogenesis and prominent vascular network.

Another effective predictor for IHD was the MAP. Davidiuk et al. first sought to create an image-based scoring system predicting intraoperative adherent perinephric fat (APF) during robotic partial nephrectomy (8). A large body of evidence noted that APF, the so-called toxic fat, is a risk factor of surgical outcomes of partial nephrectomy (PN), such as operative time, blood loss, and rates of conversion, but not associated with postoperative complications (13–15). It is not an uncommon phenomenon that the encountered sticky fat renders the surgical resection of the pheochromocytoma more difficult, resulting in hypertensive episodes and/or tachycardia as well as temporary cessation of tumor manipulation.

Our analysis also implied that necrosis is a predictor for IHD in patients with pheochromocytoma. Studies investigating the relationship of necrotic changes on CT and IHD are scarce, and contemporary series were designed to evaluate CT features in the diagnosis and prognosis of pheochromocytomas. Andreoni et al. reported that patients with cystic pheochromocytomas have more chance of being asymptomatic and having negative biochemical evaluations (16). However, Motta-Ramirez et al. reported that none of radiologic parameters including necrosis were found to differentiate incidental and symptomatic pheochromocytomas (17). Besides, Bai et al. excluded tumor necrosis as a risk predictor for IHD (7). It has been suggested that larger lesions are usually heterogeneous because of tissue necrosis or internal hemorrhage (18, 19).

With respect to the association of BMI with morbidity, the population-based findings could not be applied in patients with pheochromocytoma. Khan et al. noted that higher BMI was associated with significantly increased risk of developing CVD as well as cardiovascular morbidity (20). In addition, a meta-analysis of Mendelian randomized studies confirmed the association of obesity measured using BMI with type 2 diabetes and coronary artery disease (21). However, previous studies found that low BMI served as an independent risk factor for cardiovascular morbidity as well as severer morbidity in patients after pheochromocytoma surgery (22). Besides, our study showed that low BMI was a significant predictor for IHD, confirming earlier reports (7).

For laterality, the right-sided pheochromocytomas were associated with a higher risk of IHD than the left-sided in our study. Amar et al. noted that right-sided tumors were more common and larger, excreted greater amounts of metanephrines, and presented more frequently as incidentalomas than the left-sided (23). In addition, the shorter course and more common variation of the right suprarenal vein may render the surgery for right-sided tumor more vulnerable to substantial hemodynamic perturbations (24).

Our study found that incidence of IHD was higher in open adrenalectomy compared to minimal invasive adrenalectomy including laparoscopic and robot-assisted surgeries. This was consistent with a recent published meta-analysis of 9 studies by Li et al. (25). Open resection for large pheochromocytomas was strongly recommended in the 2014 guidelines of the Endocrine Society (26). Laparoscopic adrenalectomy including the transperitoneal approach and retroperitoneal approach has become a safe and standard technique for treatment of benign adrenal neoplasms (26–30). To be noted, Shiraishi et al. reported that the retroperitoneal approach was superior to the transperitoneal approach for right-sided large pheochromocytomas in terms of operation time, pneumoperitoneum time, and estimated blood loss. The superior perioperative outcomes of the retroperitoneal approach over the transperitoneal approach for patients with PCC were suggested from a meta-analysis of four retrospective studies (31).

This nomogram comprised of both preoperative clinical and CT risk factors could contribute to optimize treatment strategies by quantifying the probability of IHD before pheochromocytoma surgery. Once identified as a group with high risk of IHD based on the nomogram, patients should refer to a tertiary-care hospital and receive treatment from the experienced surgical team; besides, admission to intensive care unit after operation may be necessary in order to reduce the risk of postoperative complications.

Our study had several limitations in addition to those inherent in the retrospective design. Firstly, the sample size of this study is relatively small partly because we excluded a number of patients with only unenhanced CT scans. Secondly, due to a decade interval, methods of detecting catecholamines and metanephrines were not constant. Thirdly, this nomogram was developed from the cohort at a single center without external validation and adopted relatively simple parameters. Based on the uniform and complete radiological data of pheochromocytomas, further research on radiomics is needed.

## Conclusion

In conclusion, our nomogram based on clinical and CT parameters could facilitate the treatment strategy according to assessment of the risk of IHD in patients with pheochromocytoma. In addition, this nomogram needs external validation in a larger cohort.

## Data Availability Statement

The raw data supporting the conclusions of this article will be made available by the authors, without undue reservation.

## Ethics Statement

The studies involving human participants were reviewed and approved by the Institutional Review Board at the First Affiliated Hospital, Sun Yat-sen University (Guangdong, China). Written informed consent to participate in this study was provided by the participants’ legal guardian/next of kin.

## Author Contributions

Conceptualization: FZ and YY. Data collection: ZZ, JY. Methodology: CL. CT analysis: WP, YG. Formal analysis and investigation: ZZ, CL, JY. Resources: SL, FZ. Writing—original draft preparation: ZZ. Writing—review and editing: FZ, YY. Visualization, CL, SJ. Supervision: FZ, YY. Project administration: FZ. All authors contributed to the article and approved the submitted version.

## Conflict of Interest

The authors declare that the research was conducted in the absence of any commercial or financial relationships that could be construed as a potential conflict of interest.

## Publisher’s Note

All claims expressed in this article are solely those of the authors and do not necessarily represent those of their affiliated organizations, or those of the publisher, the editors and the reviewers. Any product that may be evaluated in this article, or claim that may be made by its manufacturer, is not guaranteed or endorsed by the publisher.
