# Supplementary material for: A Nomogram for Predicting Intraoperative Hemodynamic Instability in Patients With Pheochromocytoma
Source: Front Endocrinol (Lausanne). 2022 Jan 6;12:787786. doi: 10.3389/fendo.2021.787786 (PMC8772031; doi:10.3389/fendo.2021.787786)
Supplement: Supplementary file 1 [file DataSheet_1.docx]

Supplementary Material

## Supplementary Figures


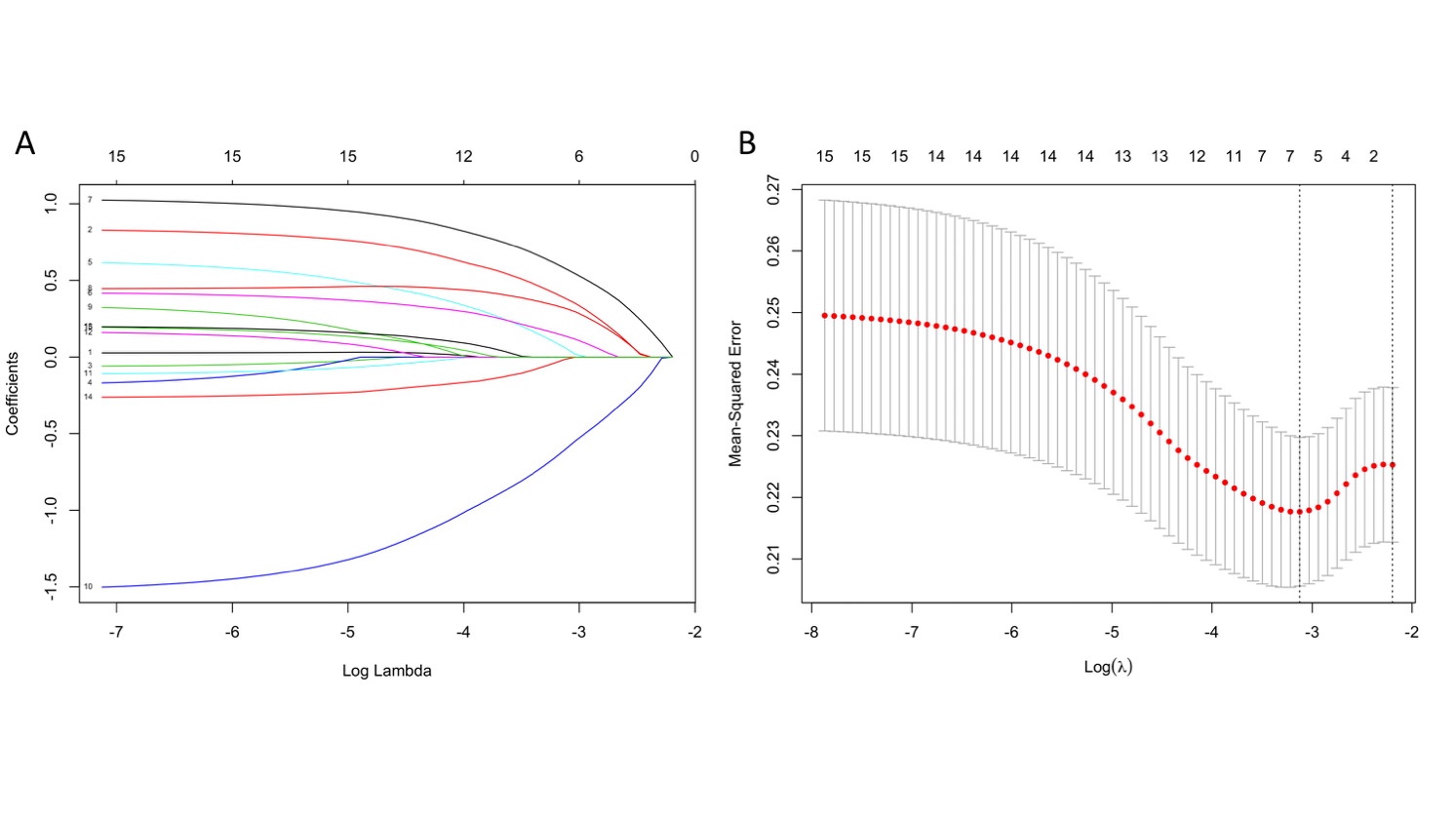


**Figure 1** Texture feature selection using the least absolute shrinkage and selection operator (LASSO) regression model. **(A)** LASSO coefficient profiles of the 7 texture features. **(B)** Tuning parameter (λ) in the LASSO model was chosen by using 10-fold cross-validation via minimum criteria.


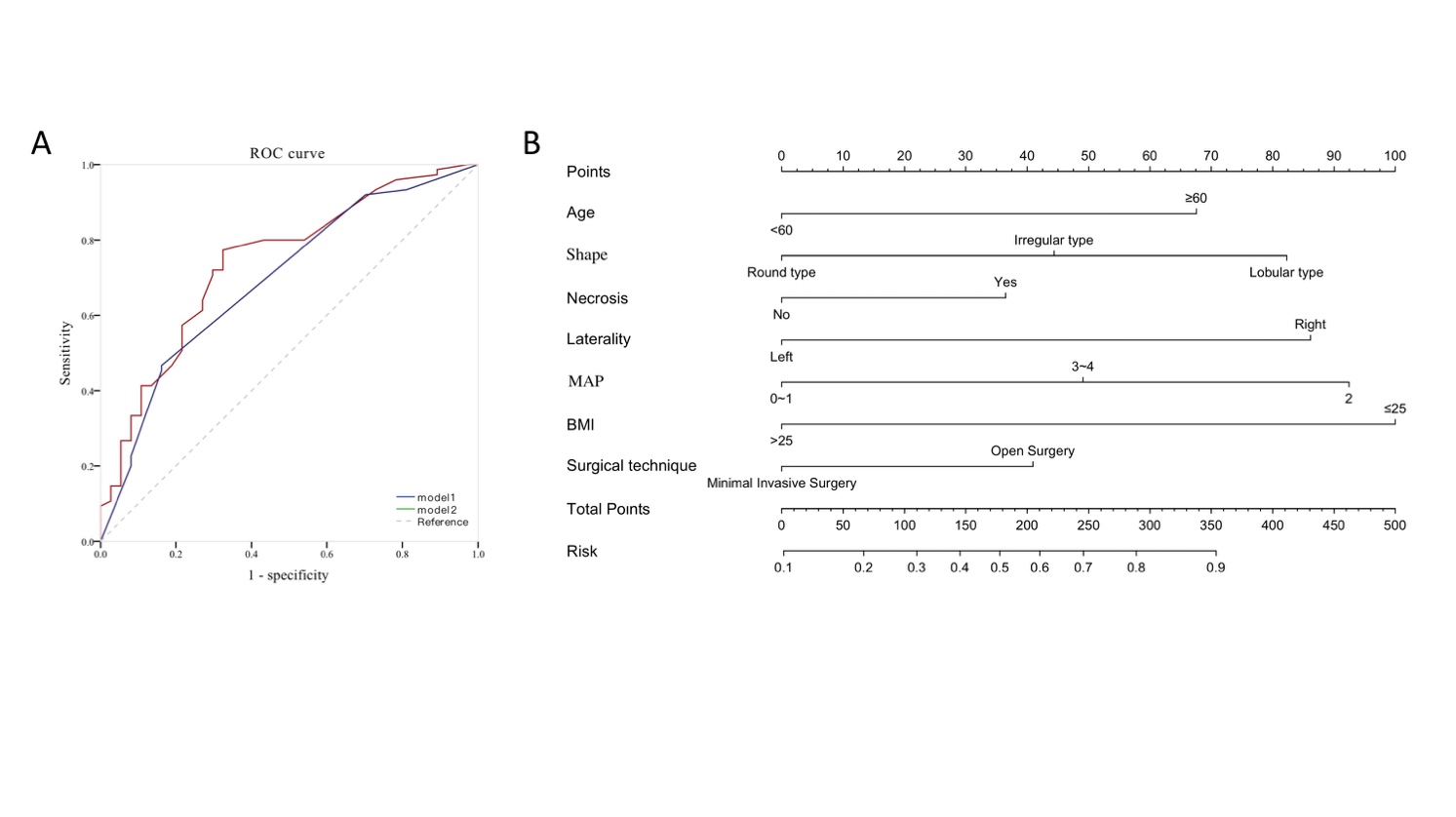


**Figure 2** Receiver operating characteristic (ROC) curves for the models and the nomogram for predicting intraoperative hemodynamic instability (IHD). (A) ROC curves demonstrating the discrimination of the nomogram with (model 1) or without (model 2) the inclusion of CT parameters. (B) Nomogram to predict IHD based on age, tumor shape, Mayo Adhesive Probability score, laterality, necrosis, body mass index, and surgical technique.


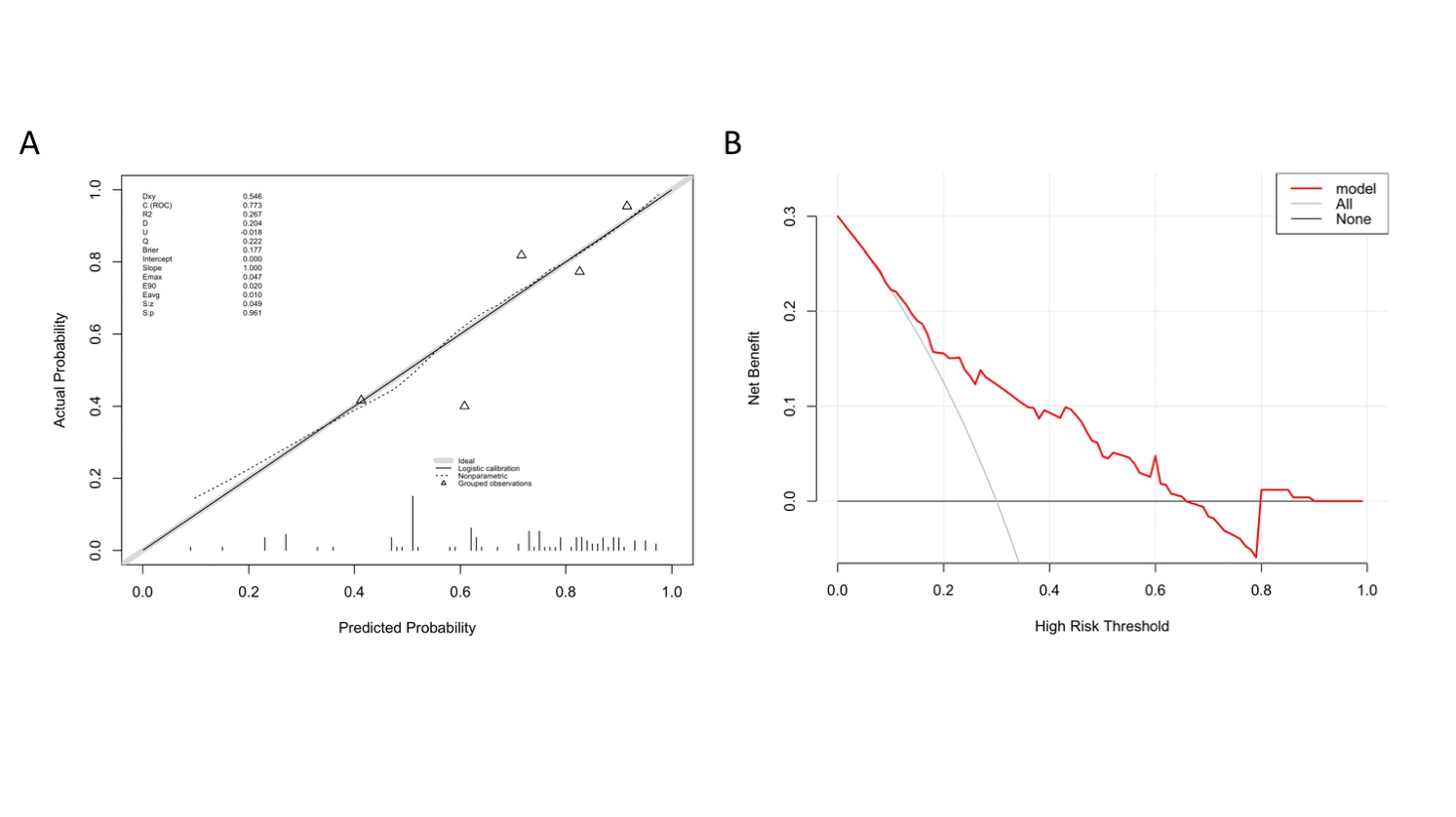


**Figure 3** The calibration plot of the nomogram and decision curve analysis. **(A)** Calibration of the nomogram to predict IHD in patients with pheochromocytomas (PCCs). The predicted probability of IHD is plotted on x-axis and the actual probability of IHD is shown on the y-axis. **(B)** Decision curve analysis shows the net benefit associated with the use of the nomogram.

## Supplementary Tables

**Supplementary Table S1.** Biochemical characteristics of patients with and without IHD

| **Levels of CAs and their metabolites** | **All patients** | **Without IHD** | **With IHD** |
| --- | --- | --- | --- |
| Elevated levels of CAs and their metabolites | 80 | 19 | 61 |
| >2 times the upper limit of the normal range of MN | 31 | 3 | 28 |
| >2 times the upper limit of the normal range of NMN | 39 | 9 | 30 |
| Normal | 21 | 11 | 10 |
| NA | 11 | 7 | 4 |

*IHD* intraoperative hemodynamic instability, *CAs* catecholamines, *NA* not available, *MN* plasma free metanephrine, *NMN* plasma free normetanephrine

**Supplementary Table S2.** C-index for each preoperative parameter predicting IHD

| **Parameters** | **C-index** |
| --- | --- |
| Age | 0.579 |
| Mayo Adhesive Probability score | 0.596 |
| Shape | 0.565 |
| Necrosis | 0.598 |
| BMI | 0.589 |
| Laterality | 0.625 |
| Surgical technique | 0.559 |

*IHD* intraoperative hemodynamic instability, *BMI* body mass index.
